# Not all biases are bad: equitable and inequitable biases in machine learning and radiology

**DOI:** 10.1186/s13244-020-00955-7

**Published:** 2021-02-10

**Authors:** Mirjam Pot, Nathalie Kieusseyan, Barbara Prainsack

**Affiliations:** 1 Department of Political Science, University of Vienna, Austria, Universitätsstraße 7, 1100 Wien, Austria; 2 OLEA MEDICAL, 93 Ave. du Sorbiers, 13600 La Ciotat, France; 3 Department of Global Health and Social Medicine, King’s College London, London, UK

**Keywords:** Radiology, Machine learning, Equity, Bias, Ethics

## Abstract

The application of machine learning (ML) technologies in medicine generally but also in radiology more specifically is hoped to improve clinical processes and the provision of healthcare. A central motivation in this regard is to advance patient treatment by reducing human error and increasing the accuracy of prognosis, diagnosis and therapy decisions. There is, however, also increasing awareness about bias in ML technologies and its potentially harmful consequences. Biases refer to systematic distortions of datasets, algorithms, or human decision making. These systematic distortions are understood to have negative effects on the quality of an outcome in terms of accuracy, fairness, or transparency. But biases are not only a technical problem that requires a technical solution. Because they often also have a social dimension, the ‘distorted’ outcomes they yield often have implications for equity. This paper assesses different types of biases that can emerge within applications of ML in radiology, and discusses in what cases such biases are problematic. Drawing upon theories of equity in healthcare, we argue that while some biases are harmful and should be acted upon, others might be unproblematic and even desirable—exactly because they can contribute to overcome inequities.

## Key points

- Many hope that the use of machine learning technologies in radiology will reduce error and bias stemming from humans.
- Machine learning technologies can, however, also exacerbate the effects of both cognitive and data bias, and pose the risk of new biases, such as automation bias.
- Some biases have harmful consequences for some groups of patients and are unjust. But this does not apply to all biases: In certain cases, the creation of deliberate bias in datasets, for example, can make decisions emerging from machine learning technologies more equitable.

## Introduction: ML and bias in healthcare

The year 1895 inaugurated the era of radiology with the discovery of X-rays by Wilhelm Röntgen. In contrast to practitioners of many other medical specialties, radiologists have always relied on machines to diagnose diseases. But these machines have changed greatly: in only a little more than a century, technological practices within radiology have evolved from anatomical image inspection to parametric images to functional and molecular imaging. The adoption of machine learning (ML) technologies—understood broadly as algorithms that advance automatically through experience—are only the latest step in this process. It was made possible by an increase in computing power and large scale data collection in many areas of healthcare, resulting from the progressing datafication (i.e., the recording of data from practices that were previously not recorded) and the digitisation of exams, as well as the emergence of digital patient management systems. Improvements in imaging systems have led to an exponential growth of imaging data that contributed to the emergence of radiomics, and have provided a fertile ground for the development of ML technologies [1].

In medicine and healthcare generally, the analysis of big data and the use of ML technologies is expected to contribute to earlier detection or even prediction of disease, more accurate diagnosis, and “personalised” decision-making about treatments [2]. In radiology, ML technologies are expected to exhibit better diagnostic sensitivity, specificity, and accuracy than previous decision-support technologies, contributing to higher rates of detection and better characterisation of disease. Some authors argue that the performance gap between humans and machines in radiology is to be particularly stark: not only can computer algorithms analyse images much more quickly than human radiologists, but they have also been shown to outperform humans in pattern recognition and in computing patterns into disease diagnoses [3, 4]. Although the specific ways in which ML technologies will impact the practice of radiology depends on the particular area of the practice [5], in one way or another they will provide support to all radiologists in different steps of the diagnostic process. They will have a substantive impact on professional practice [6], often in combination with human decision making (such as in human-in-the-loop models; [7, 8]).

Some scientists and practitioners hope that ML technologies will curb negative healthcare outcomes due to human error, such as, for example, availability bias in health professionals. Such availability bias occurs, for example, when doctors’ diagnoses are biased by what they see more often in their specific patient population, which may be older or younger than average, or more prone to specific health problems [9]. At the same time, doctors and researchers also increasingly recognise that the use of ML in healthcare can also increase certain biases. For example, the so-called GIGO (“garbage in, garbage out”) problem refers to the fact that any ML algorithm will only be as good as the data that it is trained with; if there are biases in the training datasets then these will be reiterated—and possibly exacerbated—by the ML application. Bias, therefore, has come to play a prominent role in the discussion around the implementation of ML technologies in healthcare more broadly.

### What is a bias?

Generally speaking, in medical research, bias refers to “a feature of the design of a study, or the execution of a study, or the analysis of the data from a study, that makes evidence misleading” [10, p. 104]. In clinical research studies, for example, biases could lead to results that overstate the effectiveness of a therapy. But biases also occur in medical practice where they take the form of open or implicit prejudices or errors in human reasoning that can influence the diagnostic process and health professionals’ decision-making more generally [9, 11].

It is important to emphasise that biases in ML are not merely technological problems that can be solved by technological means. For example, the composition of patient dataset used for research in imaging (e.g. population imaging) is influenced by who has access to radiology services in the first place. In many countries, there are significant differences in access to healthcare among social groups [12]. The people who do have the best access to radiology services are most likely the ones most benefitting from the application of the ML technology, because they were represented in the algorithm’s training data. While many radiologist are well aware of how biases can influence the diagnostic process, the integration of ML technologies into this field poses new challenges in dealing with biases also in the context of equity. The composition of training data for algorithms, is but one aspect why the question of equity is relevant to the implementation of ML technologies in healthcare.

There are multiple aspects to biases that cannot be adequately understood and addressed by pledges to more “awareness”. We propose to understand bias as, first and foremost, a social problem and analyse its causes and implications through a framework of equity in healthcare. This framework is also helpful to distinguish between problematic and unproblematic biases. While we think it is important to correct for obviously biased algorithms that produce inequitable outcomes, we do not consider all types of biases equally problematic. Instead of automatically assuming that all biases are “bad”, we propose to think of some biases as “good” and desirable, because they can help to overcome existing inequities in healthcare. In the following, we present our conceptual framework, and then discuss different types of biases in connection with ML in radiology. We explain in what cases these biases are problematic, and when they are not.

## Inequality vs. inequity in healthcare

In contrast to inequality, which only captures *differences* between two people or groups in terms of health outcomes or other relevant factors or characteristics, inequity is a normative concept. Inequity refers to “those inequalities in health that are deemed to be unfair or stemming from some form of injustice” [13, p. 647]. For example, while a difference in life expectancy between two groups of people that is due to one group’s propensity to engage in risky sports while the other group prefers hiking is an inequality but not an inequity, a difference in life expectancy due to unequal access to clean water and air, or unequal access to healthcare, would be an inequity. Because inequities are, by definition, always unjust, this raises the question in which cases we can speak of injustices in health. To answer this question, we consider two concepts of justice, distributive and relational justice.

Distributive justice is concerned with the fair distribution of goods. Not only is health*care* such as good, but so is health itself. Health inequalities can be unjust in a distributive sense in two ways [14]. First, inequalities are unjust if they have unjust social causes. This means that health inequalities are unjust if they correlate with an unfair distribution of other goods, such as income and wealth, for example. Adding to the example that we gave above, if the average health status in a group of 20-year-olds is better than a group of 80-year-olds, this is an inequality, but it is not necessarily unjust—insofar as it can be explained by the biological process of aging. It would be unjust, however, if the worse health outcome of older people was due to worse healthcare received by elderly cohorts in a given country. If the same difference in health status was found between the richest and the poorest 5% of the population in the same city, then this disparity would clearly be an inequity, and not merely an inequality. (This is the case if we agree that wealthier people do not have the moral right to better health than poor people, and if we assume that “unhealthy behaviours” such as smoking or bad diets are a result of deprivation, not a moral deficiency.) Second, inequalities are unjust if we, as a society, have the power to intervene upon them, but we neglect or refuse to do so, independently of whether the inequalities have a social cause or not. Some health inequalities are not unjust in themselves, because they exist due to chance, such as one person in a group of people playing soccer breaking a leg. These inequalities are, however, unjust if we fail to act upon them, for example, by failing to provide healthcare to the person with the broken leg.

While distributive justice is concerned with health outcomes and their equitable distribution within a population, relational justice is concerned with *how* healthcare is provided, and whether people are treated respectfully concerning health issues more generally. Approaches of distributive justice focus “not on distributions as inherently important but instead on the quality of social relations among citizens and/or the ways in which social institutions ‘treat’ citizens” [15, p. 204]. Whether we can speak of relational injustice, therefore, depends on the attitudes institutions and individuals express towards people as well as whether they fail to adhere to the principles of equal and respectful treatment. An example for relational injustice would be healthcare professionals treating overweight people as morally deficient because of their alleged self-indulgence and lack of perseverance. In healthcare, adhering to a relational justice perspective also means to take seriously the experiences of different patient groups and people and not to sideline patients’ concerns as irrational (as many clinicians know, sometimes a seemingly irrational fear articulates a different, very well founded concern).

The concept of relational justice is particularly helpful to analyse issues such as paternalism and stigmatisation in medicine and healthcare; issues that are difficult to address with a distributive framework. Importantly, however, from a relational point of view, unequal distributions of health are only unjust, if they are caused by or if they lead to debasement. In our understanding, to comprehensively assess inequities in health and healthcare, both the distributive and relational dimensions have to be considered. Taken together, they can help us to understand in which ways biases in radiology are inequitable nor not.

## What biases are inequitable?

Radiology as a discipline has a long history of dealing with human and machine biases and thinking about how to overcome them [16]. Cognitive biases—that is, systematic human error in image perception and interpretation—have been an issue of debate at least since the end of the 1940s [17]. Cognitive bias exists, for example, when healthcare practitioners’ past experiences unduly influence current image interpretation, or if they rely too strongly on evidence that is readily available, instead of asking what evidence is missing to make an accurate diagnosis [9, 18]. Typically, cognitive biases are described as lapses in medical reasoning. Suggestions on how to mitigate them have included measures such as bias awareness training, having several people read the same images, or reducing radiologists’ workload or distractions [17]. Authors have identified up to ten different kinds of cognitive biases relevant for radiology [9, 18]. Not all of them can be sufficiently understood by merely defining them as lapses in reasoning. In particular, to understand what it specifically is that is misjudged, overlooked or overrated and why this is the case, we have to turn to social factors such as cultural stereotypes and prejudices.

Attribution bias is a case in point of how cognitive biases are tightly connected with social categories and the meanings they carry. Attribution bias means that an attribute of a patient, such as her age, gender or race, unduly influences the diagnosis although there is no functional relationship between the social category and the clinical marker of outcome in question. Take the example of race. The terms “black” and “white” are not neutral, descriptive categories to classify people. Instead, they carry a particular cultural meaning and are intrinsically entwined with the history of racism. This means that what healthcare professionals associate with the labels of “black” and “white” influence—both consciously and unconsciously—how patients belonging to these categories are treated. Knowing an attribute of a patient, such as race, can affect healthcare professionals’ reasoning when interpreting her test results or making treatment-decisions. When race, for example, plays a role in clinical practice that is not justified by a scientifically validated  association between race and a clinical factor (e.g., patients of certain ethnicities are known to have higher risk for certain diseases) then we speak of attribution bias. A study on mammography screening, for example, has shown that radiologists were less likely to detect malignant breast lesions in patients with a minority ethnic background or low income [19]. The higher rate of false negatives in these patients suggests that health inequities are not only due to these groups’ diminished access to services but are also influenced by radiologists’ stereotypes and prejudices [12].

The problem of cognitive bias in diagnosis and decision-making is not a new one; in the age of digital health, however, cognitive biases can find their ways into datasets and potentially get “automated” through ML technologies. This, for example, can occur when the labelling of images is influenced by cognitive biases. The quality of an image—a single data item—depends, on the quality of imaging technologies and their correct use. Ideally, images have a proper resolution and are free of artefacts. But good-quality images also need to be labelled correctly. In order for an algorithm to accurately detect a disease in question, the training database has to be composed of properly labelled images—at least in supervised and semi-supervised ML. A misleading labelling or error of delineation of the organ increases the inaccuracy of an algorithm. This is sometimes referred to as misclassification or measurement error [20]. While image quality is partly a technological challenge, there is also a social component to it.

As we have pointed out, culturally influenced cognitive biases about people or groups can have an impact on what radiologists see and what they do not see in images. This means that through the interpretation and labelling of images, radiologists’ cognitive biases can translate into data biases. Healthcare data more generally is likely to reflect the—often unconscious—discrimination of certain groups of patients along the line of socio-economic status, gender, race, and other social categories. Datasets are biased in a qualitative way if they include data about misdiagnoses, and these misdiagnoses are structural, because they particularly affect patients belonging to a specific social group, such as the socio-economically disadvantaged, women or racial minorities. In turn, an algorithm developed with this data, is more likely not to correctly detect disease in the populations that are prone to misdiagnosis.

Proponents of the application of ML algorithms in healthcare often have cognitive biases on the side of healthcare professionals in mind when they hope for technology to overcome biases. They often overlook that, as long as humans hold cognitive biases, these biases will likely also shape practices of data generation and ultimately data itself. Furthermore, ML algorithms are built by human developers; knowingly or unknowingly, their way of thinking and the biases they hold can influence and shape the technologies they are building. This means that the same cognitive biases that influence radiological reasoning and decision-making can influence developers and therefore the structure of an algorithm. This might happen, for example, if the developer is influenced by her previous experiences in the sense that if she has already developed similar technologies, she might programme in such a way that the new technology matches her previous results. In particular, decisions about whether certain variables should be included or excluded from an algorithm and how they are weighted are prone to programmers’ biases [21]. Computer science is only starting to acknowledge that cognitive biases on the side of programmers can have an impact on the machines they develop. Recently, Pedersen and colleagues [22] have empirically shown that the context in which programmers are socialised influences the technologies they build. This means that developers’ cognitive biases can translate into machine biases.

### Are cognitive biases inequitable?

Radiologists and their reasoning can be biased, such as when they diagnose and treat people from particular social, ethnic or religious groups differently from other patients. Such biases are then also likely to be reflected in healthcare data, such as in how images are labelled. Think, for example, of the higher rate of missed detections of breast cancer in images from women belonging to ethnic minorities [19]. If this data is used to build decision-support technologies, the cognitive bias present in clinical practice might be reiterated and reinforced. Explicit and implicit forms of discrimination in healthcare and its datafication and technological perpetuation are a form of relational injustice, because equally respectful treatment among patients is not ensured. At the same time, such—often unconscious—discriminations can have distributional consequences such as women from ethnic minorities not receiving cancer treatment in time.

Not only healthcare professionals, but also software developers can be prone to cognitive biases, which can get inscribed in technologies they build [22]. This is not a moral deficiency or a characteristic of certain professional groups, however: Because humans are social beings our experiences and our consciousness are necessarily influenced by society and our position within it. Our perceptions and experiences are shaped by a multitude of dimensions of which class, gender, and race are merely the most visible ones. That we are all biased does not mean, however, that we do not have to take responsibility for our blind spots and try to overcome the implicit biases that we are aware of: the motto of “fairness through awareness” means that critical scrutiny of one’s own implicit biases is the first step to being able to prevent discriminatory practice. We have the moral obligation to mitigate the harmful consequences also of implicit and unconscious bias, and to avoid structural discrimination. Awareness, however, is not enough precisely because our own biases are often not visible and accessible to us. From a perspective of relational justice, therefore, it is important to include a wide range of experiences and perspectives in the process of generating data and developing technologies. This means that we need to ask who is generating data, analysing datasets and building technologies, and for whose benefits—both in radiology and computer science. For example, radiology, like medicine in general, has been a domain in which women and minorities have traditionally been underrepresented, and they still are [23, 24]. Also computer science remains a male-dominated field [25]. The gendered culture of computer science, which is furthermore characterised by a strong belief that any issue can be solved with better technologies, increases the risk of certain biases going unnoticed [26], such as biases that disadvantage women [27].

While some cognitive biases are shaped by social and cultural factors, others pertain to how humans process information. Culturally influenced cognitive biases on the one hand are inequitable if they could have been avoided—e.g., by actively working towards greater diversity among radiologists, software developers, and decision-makers at healthcare institutions. On the other hand, cognitive biases pertaining to how humans process information can also be inequitable if mitigating actions, such as the reduction of workload and stress are not taken. Additionally, from a perspective of relational justice, it is important that people—be they patients, radiologists, programmers, or others—who express concerns about potential bias in ML technologies are taken seriously and treated respectfully. This also pertains to how potential mistakes by radiologists are dealt with. Independently from whether they work with ML algorithms or not, because “[a] just culture in which patient safety is emphasised in conjunction with respect for individual physician personal worth is requisite for any error-reduction program to be successful in practice “ [17, p. 837].

### Qualitative vs. quantitative biases

We have shown that a problematic labelling of images can lead to *qualitatively* biased datasets, biased ML technologies and ultimately inequitable outcomes. ML technologies, however, can also be biased because of *quantitative* misrepresentations in datasets. In such cases, every single data item can be of high quality (e.g., every image is correctly labelled), but the dataset as a whole can still be skewed. This happens, for example, when an image dataset does not adequately represent all patient sub-populations in terms of numbers. Quantitative misrepresentation is not a new problem in medical research and practice, of course: in the context of clinical trials, for example, the systematic underrepresentation of the elderly, ethnic minorities or women is a well-known problem and has attracted criticism [28, 29]. In clinical trials, such underrepresentation of specific groups is a problem when results of research is generalised even though a tested treatment might be less effective or less safe for the populations that were not included in the trial. Similarly, the quantitative misrepresentation of groups in datasets used for research in imaging could do harm when it leads to algorithms or decision aids that do not “fit” the groups that were underrepresented.

In contrast to clinical trials data, digital health data is often collected in routine healthcare situations. This means that biases concerning the representation of specific groups in such datasets are often difficult to detect—also because they are entangled with a variety of social and economic factors. Regarding data stemming from electronic health records, Prosperi and colleagues [30, p. 10] emphasised that such data is “inherently biased by the patient population structure, frequency of healthcare visits, diagnostic criteria, and care pathways.” This also applies to imaging data that is collected in everyday healthcare settings. Glover and colleagues [31], in a study on missed imaging appointments in the United States, found that racial minorities and people with low socioeconomic status missed more appointments than other groups. The authors attribute these results to multiple factors including those groups’ limited financial resources and geographic barriers, which make it more difficult to make appointments. Missed radiology appointments are associated with a later detection of disease and therefore higher mortality. The disadvantage of these groups, however, is even further exacerbated by the fact that they are underrepresented in the imaging datasets of their hospital or healthcare provider and an algorithm developed with this data might be less accurate for patients from these groups.

### Is quantitative misrepresentation always inequitable?

Who is represented in imaging datasets in quantitative terms is shaped by the access different people and groups have to healthcare. The distribution of resources such as income and wealth influence access to healthcare decisively. Often, socioeconomic factors are entangled with other categories, such as race or gender. Differences among socioeconomic groups in their access to healthcare are an inequity because their cause is socially unjust. If access to care is inequitable, an algorithm based on data collected in healthcare settings could be seen as inequitable as well. This is because people with restricted access to healthcare are “missing” in the dataset and the decisions the algorithm suggests will likely be less adequate for those groups missing. Quantitatively biased datasets can therefore lead to differences in the quality of healthcare people from various social groups receive, and different social groups receiving unequal quality of healthcare is unjust.

### When is a dataset unbiased?

Similar to the difficulty to define what cognitively unbiased humans are, however, it is not always clear what an unbiased dataset would look like. Is a dataset unbiased when it adequately represents all populations that are treated at the hospital for which the software is developed? Is it unbiased only when it adequately represents the wider population in the local area (because the hospital may already be “biased” in that it treats mostly wealthy patients, for example)? There are no right and wrong answers to these questions if they are asked in the abstract. They always need to be answered within a specific context; considering also issues of qualitative bias in combination with quantitative bias. Consider the case of rare diseases: all people with a particular rare disease X could be perfectly represented in a dataset and yet the people with this disease might receive lower-quality healthcare than people with common diseases. This is because the group of people with rare disease X might be so small that the amount of data about them is not sufficient to build an algorithm that produces reliable results. People with the rare disease X receiving lower-quality healthcare due to the small number of people suffering from the disease, however, can still be inequitable. In this case, the inequity occurs if we had the power to correct for the unjust differences in healthcare quality but failed to do so.

This means that efforts to ensure the best possible diversity of patient data are essential for the development of equitable ML technologies even if this includes a departure from the ideal of quantitative representativity. Special concern must be given to the improvement of databases by detecting omissions and filling potential data gaps. Furthermore, in cases of low-prevalence conditions, developers have to account for rare diseases and the small quantity of data available on them and adjust algorithms accordingly. Referring to cancer imaging, Bi and colleagues [32, p. 149] stress that “[a]lthough data curation and modelling practices are biased in nature, because they take into account specific patient cohorts, a conscious effort must be put into understanding exactly who will be the ultimate beneficiaries and stakeholders of such technology.”

At the same time, besides filling data gaps or adapting algorithms to attenuate the potentially negative consequences of inequitable biases, it may be necessary in some cases to create biases on purpose. For example, if data from underserved populations—such as economically deprived groups—are oversampled to compensate for a previous invisibility of these groups, then the data collection has a deliberate bias that seeks to create a beneficial effect, namely to prominently include a group that had previously been marginalised. Another example would be to deliberately over-sample  people with darker skin for a dataset training an algorithm detecting skin diseases as certain colour contrasts may be less easy to discern on dark skin than on light skin. In these cases, biases are explicitly equitable. They would be inequitable if we knew about the discriminatory effect of an algorithm—due to social factors or not—but had failed to take action against those outcomes (Table 1).

**Table 1:** Different types of bias in healthcare (authors’ depiction; see also [20, 33])

Type of bias	Bias is rooted in	Example	Effects
Data bias	Low quality of datasets, due to		All types of biases can translate into machine biases and have effects on equity, accountability, and transparency
	(1) quantitative misrepresentation of certain patient groups in datasets and/or	A set of training data for machine learning includes images of only (or mostly) males	
	(2) qualitative misrepresentation of certain patient groups in datasets (e.g. wrong labeling of images)	A set of training data for machine learning includes images of socio-economically disadvantaged women as “healthy” controls, although some of them have diseases that were not diagnosed	
Cognitive bias	Features in the human processing of knowledge and cultural factors	Radiologists’ culturally informed stereotypes lead to a high rate of false negatives in breast images of socio-economically disadvantaged women	

Although radiologists have been aware of certain biases for a long time, the implementation of diagnostic ML algorithms and the potential translation of cognitive and data biases into these technologies raises the problem of bias to a new level. This is because a ML algorithm has the capability to “autonomously” identify patterns and continuously readjust its own decision making on the basis of what it has “learned”. This can lead to the identification of associations between phenomena or factors that were not known to be correlated (and that may not be connected through a causal link). While learning about new associations can positively impact patient care, acting upon associations discerned through data mining also bears the risk of increasing bias, especially if the underlying pathway or dynamic that accounts for associations is not known. This might occur, for example, when a diagnostic algorithm correctly identifies a portion of the lung on a chest x-ray as abnormal where humans have failed to determine this. In this case, the very advantage of using diagnostic ML technologies, namely their capacity to detect patterns in images that are not visible to the human eye, might create a dilemma if it cannot be determined how the algorithm reached its conclusion. It might well be that the algorithm correctly marked the tissue as abnormal (as determined by comparison with confirmed outcomes), but as long as long as this has not been established, healthcare decisions should not be based solely on the decision of the algorithm [17].

## New challenges posed by ML: dual valence, and automation bias

Problems due to cognitive or data biases in the context of ML technologies might be further intensified if there is a “dual valence” problem. A dual valence problem exists if a seemingly innocuous characteristic included in decision making—such as someone’s postcode—correlates with characteristics that denote stigmatised or discriminated groups [34]. For example, as Harald Schmidt [35] recently argued, if triage decisions in a pandemic situation consider a person’s overall health status and expected benefit—which seems like a legitimate thing to do when deciding who gets a respirator, for example—then this decision already has social and economic discrimination “baked in”. This is because certain groups, often minorities, have worse health status due to the disadvantages that they have suffered. In other words, “health status” has dual valence because it correlates with social and economic disadvantage. In other words, as tempting as it may seem to use ML to identify new patterns that could improve patient care, the risk of bias in the sense of undue discrimination does not disappear if decisions are left to machines.

Generally, dealing with biases in ML technologies is not only a challenge because problems such as dual valence situations  are often hard to detect but also because of automation bias—one more kind of bias that could affect research and practice in radiology. Automation bias is a form of cognitive bias and it occurs when humans overestimate the validity or the predictive power of information produced by an automated system such as an ML algorithm. Such overreliance on technology can affect any instance where automated decision-support is applied. It occurs when healthcare workers rely on the technology so much that they refrain from applying their own critical judgement. It may be tempting for clinicians to defer to the results produced by the machine because the machine seems more trustworthy, “safer” for the person to rely on, or less biased than human action [36–38]. This problem does not go away when policies or protocols insist that machines merely indicate possible results, but humans remain the decision makers: also in these cases, there can be social or other pressures on people to go by what the machine suggests. Moreover, automation can “undermine the epistemic authority of clinicians” [39, p. 1]—even for themselves. Regarding the interpretation of mammograms, for example, Povyakalo and colleagues [40] found that while automated support had beneficial effects on the detections of radiologists with less advanced interpretation skills, it had a detrimental effect on the detections of radiologists with advanced image interpretation skills.

Similarly, automation bias can lead to the devaluation of patients’ experiential knowledge and other factors not represented in the data an algorithm is built on [41]. If health professionals increasingly rely on automated decision-support systems, this also means that they place more emphasis on quantifiable and computable aspects of health and disease that can be captured in the form of digital patient data. If quantifiable aspects of patients’ health gain more importance, then contextual information about patients, their experiences and values necessarily take a backseat. This might pose a problem because these non-quantifiable aspects are often relevant for determining what constitutes good healthcare.

Automation bias is not only a potential hindrance in gaining awareness and dealing with other forms of biases in connection with ML technologies. The potential devaluation of human expertise and experiential knowledge also raises the question whether the use of ML algorithms in itself might be problematic or even inequitable. In this context, the question is how ML technologies are implemented in healthcare, regardless of whether they are biased or not. As we have discussed before, automation bias can occur because of excessive trust in data and technologies and the neglect of context, patients’ experiences and critical human judgement of health professionals. The use of ML algorithms can undoubtedly be helpful in many instances. But even when they are used “merely” for decision support, once algorithms and their suggestions gain a certain authority, health professionals as well as patients might experience relational injustice. This can occur if health professionals’ experiential knowledge and expertise is devalued in contrast to knowledge produced by machines.

At the same time, health professionals themselves enact relational injustice if they consider contextual information and information stemming from patients less important than the “hard data” of somatic biomarkers. For example, if doctors give more credence to an automatically suggested treatment than to the patient’s wish for another similar but slightly different treatment option. As Bennett and Keyes [42, p. 2] emphasise, “by adding technical and scientific authority to medical authority, people subject to medical contexts are not only not granted power, but are even further disempowered, with even less legitimacy given to the patient’s voice.” In such cases, patients experience injustice “for being a *patient*” [42, p. 2]. Awareness about the areas in which humans do—and will continue to do—better than machines is crucial in order to minimise relational injustices in connection with automation bias. One of these areas is the meaningful interpretation of contextual and qualitative information in healthcare settings.

Notwithstanding automation bias and the question whether the application of a ML technology is justified and just in the first place, the management of ML biases plays an important role in ensuring equity in data-driven healthcare. A justice-oriented management of technologies and potential biases includes continuous education and realistic communication to radiologists about the workings of the technology as well as its specific capabilities, limitations and risks—also regarding issues of justice. Considering potential modifications of ML algorithms this cannot be a one-off event but is to be understood as a process where radiologists are regularly informed about new developments and findings. Users should also be enabled to evaluate the outcomes of ML algorithms, to understand the everyday added value of the technology for their clinical work but also to detect and understand errors or pitfalls. This is also relevant for radiologists’ engagement with patients and their inclusion to decision-making. At the same time, radiologists’ and patients’ concerns about potential biases but also about working with ML technologies more generally or being subjected to automate decision-making should be taken seriously, thoroughly assessed, and addressed. For regulatory clearance, the possibility that ML technologies can change and biases sometimes only become visible over time means, that the quality of a ML technology has not only to be assessed once before its implementation. Instead, monitoring and performance controls have to take place regularly after the initial approval and as long as the technology is in use.

## Conclusion

In this paper we have argued that not all biases are bad: biases can be problematic and unproblematic. They are unproblematic if they contribute to greater equity (or do at least not detract from it), meaning that they are based on or create a distortion of reality that is not unjust and might even be beneficial. Biases are problematic if they are inequitable. This is the case if either they are based on or lead to the unjust distribution of goods or because they are based on or lead to the undue discrimination of certain people and social groups. Biases are unjust in a distributive sense if they lead to an unfair distribution of goods such as access to healthcare services. Such cases are particularly concerning if they exacerbate existing distributive inequities. From a relational justice perspective, ML algorithms are unjust if they are used for objectives that undermine equal respect and dignity among patients, independently of whether they are biased in a technical sense. Finally, biases may be relationally unjust if concerns about the use of algorithms or their outcomes are not being taken seriously and people’s concerns are dismissed.

In order to assess whether biases are inequitable or not points to the importance of reflecting upon the social categories that influence the generation of data, the quality of datasets as well as practices of building and applying technologies. It is not problematic as such that data and machines are influenced by social categories—this is often unavoidable. If the social condition, however, is characterised by inequities and there is a lack of awareness about them, technologies might contribute to their “automisation” [43], meaning that inequities get perpetuated and solidified on a large scale while at the same time they might get harder to detect. This points to the limits of understanding biases in algorithms as purely technical problems that can be corrected with better computational models. Instead, they prompt questions about the “logic that produces advantaged and disadvantaged subjects in the first place” [44, p. 901]. Crucially, this means taking into account societal power relations and how they influence the production and collection of data in healthcare as well as the development of data technologies and their application [44, 45]. Given its strongly data- and technology driven nature, this is particularly important for radiology.

## Data Availability

This paper reviews published research and does not use any original data.

## Abbreviation

ML: Machine learning
